# “Acknowledge my grief” - experiences and needs of bereavement support in Sweden following the death of a loved one

**DOI:** 10.1186/s12889-026-29094-2

**Published:** 2026-08-19

**Authors:** Rakel Eklund, Agnes Englund, Frida Berglund, Josefin Sveen

**Affiliations:** 1 Department of Women’s and Children’s Health, Uppsala University, Uppsala, Sweden; 2 National Centre for Disaster Psychiatry, Department of Medical Sciences, Uppsala University, Uppsala, Sweden

**Keywords:** Bereaved individuals, Bereavement, Grief, Support, Qualitative method

## Abstract

**Background:**

Grief affects most people at some point, and is commonly associated with psychological distress, reduced well-being, and difficulties in everyday functioning. Support for the bereaved is crucial for grief management, but there is a lack of evidence on how grief support should be arranged and offered. This study aims to highlight the support bereaved people in Sweden have been offered and what they wished had been offered.

**Method:**

Participants answered a web survey with open questions about which support they received after a loved one’s death, if they were satisfied with the support and what they felt was missing. A total of 472 participants’ answers were analysed with a conventional content analysis, which resulted in eleven categories divided into two overarching themes.

**Results:**

A majority (58.5%) of the participants felt that the support had not been enough. Received support varied and included support from social networks, healthcare, civil society and church, but also a lack of support. Desired support included information about grief and grief support, improved structure and organisation of given support, availability of counselling, practical help, economic assistance and wider knowledge about grief in society.

**Conclusion:**

Bereavement support in Sweden is provided by a wide range of societal factors, but the findings suggest important gaps in its structure, continuity, and visibility. Further research is needed to determine which bereaved individuals need which types of support, and when, in order to ensure that support is appropriate, effective, and socioeconomically sustainable.

## Background

 Grief is a response to loss after the death of a loved one [1, 2]. Psychological reactions of grief may vary across situations and individuals and include feelings of shock, anger, guilt, loneliness, and emotional numbness [3]. Furthermore, grief is often manifested as physiological reactions, such as a lack of energy, overactivity, crying, loss of appetite, memory difficulties, sleep disturbances, pain, and dizziness [2]. Grief is also associated with higher levels of systemic inflammation and maladaptive gene expression in immune cells [4]. There is also evidence that cortisol levels are higher in individuals who have lost a loved one compared with those who have not [5], indicating higher stress levels in the body with a resulting systemic response. These are normal and expected reactions to loss, which do not necessarily require professional support or psychological interventions [6]. Beyond these reactions, bereavement has also been linked to broader health consequences such as increased mortality during the first months after the death [2, 7].

Approximately 90,000 people die annually in Sweden [8] leaving a large number of individuals experiencing grief at any given point in time. Consequently, bereavement constitutes a recurring and widespread public health concern, indicating a substantial and continuous need for accessible support for people during grief.

In general, support interventions can be divided into those offered at or shortly after the time of death, and those offered later in bereavement, after the immediate practical and informational needs surrounding the death have passed. The most common bereavement support within healthcare is that provided immediately after the death, such as information about grief and grief reactions, practical support related to funerals or organ donation, and information about available support services [9]. Within Swedish healthcare, the initial focus is on informing the bereaved about the death, and if possible, allowing them to say farewell to the deceased [10]. In cases of unnatural deaths outside the healthcare system, the police are responsible for informing the bereaved about the death [11]. There are no national guidelines in Sweden for how bereavement support should be designed or provided to those affected. However, employees in Sweden may receive up to ten days of paid leave following the death of a close relative. If a longer leave is required, this must be arranged through individual agreements with the employer or sick leave certified by a physician [12]. In addition to healthcare, civil society, as well as family and friends, play a major role in offering bereavement support. In Sweden, the Church of Sweden assumes substantial responsibility for supporting bereaved individuals regardless of religious affiliation [13]. With increasing digitalisation, online support has also grown, both in the form of informational and supportive websites [14], support groups via social media platforms [15], and digital self-help apps [16, 17]. The amount and type of bereavement support needed differ between bereaved individuals and largely depend on the severity of grief reactions and symptoms, the circumstances surrounding the loss, and the time elapsed since the death [18]. Support for bereaved individuals can be offered at varying degrees of intensity [19], ranging from informal support in everyday life to specialised clinical treatment. Most bereaved people are in need of and manage well with the support and understanding from family and friends, fewer benefit from organised or professional support, and only a small minority require psychiatric treatment [19, 20]. For those with more severe mental health problems, such as prolonged grief disorder (PGD), psychological treatment may be indicated. With the introduction of PGD as a diagnosis in the International Classification of Diseases 11th Revision (ICD-11) and the Diagnostic and Statistical Manual of Mental Disorders, Fifth Edition, Text Revision (DSM-5-TR) [21, 22], discussions regarding general guidelines for support interventions have increased. Guidelines for bereavement support are lacking in many European countries, including Sweden, but initiatives exist to compile knowledge and attempt to develop clinical guidelines [18]. The bereavement support offered varies between countries, religions, cultures, healthcare institutions, and medical specialities, and may depend on the relationship the bereaved had to the deceased and the cause of death. The wide variation in bereavement support and the large number of bereaved individuals make it of interest to study what support is offered to the bereaved and what support bereaved individuals wish to receive. Grief reactions may have substantial consequences for bereaved individuals’ everyday functioning, wellbeing, and ability to manage work, family responsibilities, and social life. Therefore, understanding what support bereaved individuals receive, what they perceive as missing, and how support could be better organised is important for developing accessible and appropriate bereavement support in practice. There is a lack of studies investigating bereaved individuals’ experiences of bereavement support in Sweden. Therefore, the aim of this study is to contribute to making the experiences and wishes of bereaved individuals regarding support visible, thereby enabling a better understanding of how bereavement support should be designed in Sweden.

## Methods

### Design

This study is part of a cross-sectional survey examining self-reported grief, prolonged grief, and bereavement support. The present study is based on two open-ended questions concerning bereavement-related support and one closed-ended question regarding whether the support was sufficient or not. In connection with the survey responses, sociodemographic data were collected, along with information on the type of relationship to the deceased and the cause of death. The study was performed using an inductive qualitative method [23] in accordance with the Consolidated Criteria for Reporting Qualitative Research (COREQ) checklist [24].

### Participants and data collection

**Fig. 1:**
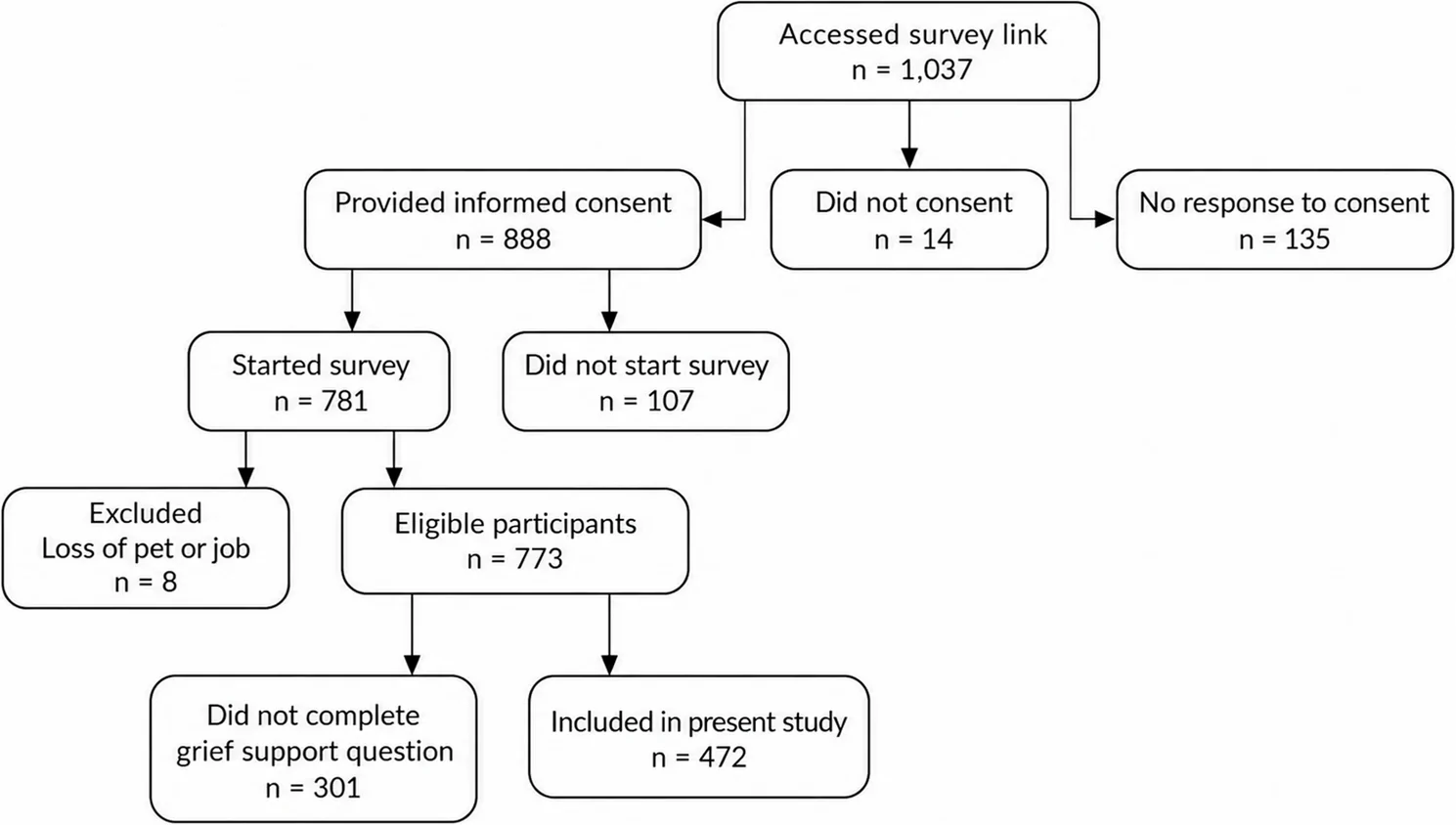
Participant flow summary

Participants 18 years and older who had lost a loved one (e.g., a family member, partner, or close friend) at least one month prior to the study were eligible to participate. No exclusion criteria were applied. Participants were recruited from August 2025 to January 2026 via social media (Facebook, Instagram, Reddit and LinkedIn) and a website associated with the research project (https://kompliceradsorg.se). A total of 1,037 individuals accessed the survey link. Of these, 888 provided informed consent to participate, 14 declined participation, and 135 did not respond to the consent question. Among those who consented, 781 proceeded to the survey, whereas 107 did not start it. Eight participants were excluded because they reported the loss of a pet or a job. Of the remaining 773 participants, 301 did not complete the question regarding grief support. Consequently, the final sample comprised 472 participants (Fig. 1). The survey was administered as a web-based questionnaire using the REDCap survey tool [25]. Survey responses in which participants had answered at least one of the questions were included in the analysis. The questions included in this analysis were: *What support have you received in your grief?*; *Have you received sufficient support?* (Yes/No); *If you wished for more support*,* what kind of support would you have wanted?*

### Data analysis

Sociodemographic data and the responses to the closed-ended question (yes/no) were analysed with descriptive statistics. Responses to the two open-ended questions were analysed using qualitative content analysis [23]. To enhance reflexivity, methodological choices and the analytic process were discussed between the researchers in the team throughout the whole process. The analysis was conducted manually. Initial coding was performed by AE using printed responses and pen and paper. Codes, subcategories, and categories were subsequently discussed and further developed by AE and RE using a whiteboard to visualise patterns, similarities, differences, and relationships within the material. All responses were read word by word, and codes were formulated and interpreted by one of the authors (AE). The codes were discussed between two of the authors (AE and RE) before the analysis proceeded. The codes were analysed one survey question at a time. The codes were sorted into clusters based on similarities and were then further divided depending on patterns, similarities, and differences. The clusters were ordered in a flowchart-like manner depending on how general or specific they were. The resulting grouping of clusters constituted subcategories, which were compared and organised into categories. The categories were then compared with one another and organised into themes. The analysis resulted in eleven categories organised into two overarching themes based on the research questions (see Table 1). To enhance the credibility of the analysis, representative quotes from different participants were selected and included in the results.

**Table 1:** Examples of the analysis process

Open-ended responses	Code	Sub-category	Category	Overarching themes
*“Only talked a little with a psychologist*,* grief groups*,* listened to music*,* read books”*	Conversation with a psychologist	Conversations with a psychologist	Professional support and treatment within healthcare	Bereaved individuals’ experiences of support received or offered
	Grief support group	Organised meetings and groups for grief support	Supportive societal functions for the individual and the family	
	Listened to music	Self-help	Active personal efforts in grief management	
	Read books			
*“Hardly anything during the past 10 years. The little support I have received has mainly come from my boyfriend; he listens and is there when I am sad.”*	Hardly anything	No support - insufficient	No support received	
	Partner who listened	Supportive significant others	Managing grief together with close family and friends	
	Supportive partner			
*“Psychotherapy*,* trauma therapy*,* and sleeping pills to take occasionally. Understanding from those around me that this is a kind of lifelong disability and that one does not have the strength one had before.”*	Psychotherapy	Therapy for grief	Access to treatments for grief and grief-related disorders	Support that the bereaved wished they had received
	Trauma therapy			
	Sleeping medication	Treatment of physical problems		
	Understanding from others that grief takes time and energy	Understanding of grief symptoms	Increased knowledge about grief in society	
*“That someone had contacted me after she died. That I would not have had to pay for all the therapy myself. That there had been more support for family members after suicide. For example*,* for her children.”*	Contact after the death	Outreach support	Organisation of support with consideration of temporal aspects	
	Subsidised therapy	Financial support	Practical support in everyday life	
	Family support based on cause of death	Specialised support based on relationship to the deceased or cause of death	Expanded bereavement support through human interaction	
*“Everything is unclear*,* information about where one can turn when it happens.”*	Information about available support	Information about support	Information for the bereaved	

### Ethical considerations

The study was approved by the Swedish Ethical Review Authority (reference number 2025-04323-01), and all study procedures were performed in accordance with the Declaration of Helsinki. Participants were asked to provide informed consent before completing the survey in REDCap. Upon completion of the survey, participants received automated feedback based on their score on the Traumatic Grief Inventory Self-Report Plus (TGI-SR+). Participants with scores indicating possible PGD were informed that they might benefit from professional support and were encouraged to contact their primary healthcare provider or seek grief-specific treatment. They also received links to an ongoing study evaluating an internet-based psychological treatment for PGD, where they could obtain further information and, if interested, enroll. Participants scoring below the cut-off were informed that, although their grief reactions did not appear to require professional care, support was available if their grief was causing significant distress. They were encouraged to contact their primary healthcare provider or seek grief counselling or treatment and were informed about bereavement support available through non-profit organisations and support groups.

### Use of AI

ChatGPT (OpenAI; GPT-5.4 Thinking) was used to assist with linguistic revision and language editing of the manuscript. All substantive content, interpretation, and final decisions were made by the authors.

## Results

### Participants and their deceased loved ones

Most participants were women (72.7%) between 25 and 54 years old (67.2%). The largest proportion of participants had completed university or college education (58.1%) and were in a committed relationship at the time of the study (54.5%). The majority were working or studying (77.5%). Time since loss ranged from < 1 to 58 years (mean: 7 years). Relationships to the deceased varied; the largest groups were those who had lost a child (22.9%), a parent (22.4%), or a partner (21.0%). Long-term illness (e.g., cancer or neurological disease) (32.8%), acute medical conditions (e.g., stroke or myocardial infarction) (26.1%), and suicide (26.1%) were the most common causes of death. For further details, see Table 2.

**Table 2:** Sociodemographic data

	*n*	%
Gender		
Women	343	72.7
Man	119	25.2
Non-binary/other	10	2.1
Age		
18–24	28	5.9
25–34	120	25.4
35–44	115	24.4
45–54	82	17.4
55–64	81	17.1
65–74	41	8.7
75–84	5	1.1
Highest completed level of education		
No completed education	3	0.6
Compulsory school	15	3.2
Upper secondary school	112	23.7
Adult education, apprenticeship programme, etc.	68	14.4
University or college	274	58.1
Current occupation		
Employed/self-employed/student	333	70.6
Retired/disability retirement	40	8.5
On sick leave	39	8.3
Unemployed	14	3.0
Parental leave	8	1.7
Home maker	2	0.4
Employed/self-employed/student and on sick leave	20	4.2
Employed/self-employed/student and unemployed	6	1.3
Employed/self-employed/student and retired/disability retirement	4	0.8
Employed/self-employed/student and parental leave	2	0.4
Employed/self-employed/student and home maker	1	0.2
Unemployed and on sick leave	1	0.2
Unemployed and retired/disability retirement	1	0.2
Unemployed and home maker	1	0.2
Current relationship status		
Married/partner/cohabitating/living apart in committed relation	257	54.5
Single	136	28.8
Divorced/widowed	77	16.3
Other	2	0.4
Relationship to the deceased		
Child	108	22.9
Parent	106	22.4
Partner	99	21.0
Sibling	73	15.5
Grandparent	41	8.7
Friend	37	7.8
Other		
Second-degree relative	5	1.1
Parent-in-law	2	0.4
Former partner	1	0.2
Cause of death		
Long-term illness (e.g., cancer, neurological disease)	155	32.8
Acute medical condition (e.g., myocardial infection, stroke)	123	26.1
Suicide	123	26.1
Accident	47	10.0
Homicide, other act of violence	8	1.7
Other		
Substance use	11	2.3
No response	3	0.6
Do not know	2	0.4

### Perceptions of whether the offered support was sufficient

The results show that 276 participants (58.5%) reported that the support they had received was insufficient, while 194 participants (41.1%) reported having received sufficient support. Of those who perceived the support as sufficient, 77 responded to the free-text question regarding what support they would have wished for, and these responses are also included in the results.

Several participants described a sense of resignation, expressing that no support could be sufficient. One participant whose child had died wrote: “*In fact*,* I should have answered no to the question*,* but I have received a great deal of support and cannot say what support I would have wanted instead. There is no way to reduce the grief after the death of a child. Acceptance is something I will never reach.*” (Parent to the deceased, 65–74 years). Another participant described realising that the support had been sufficient in retrospect: “*At the time*,* I probably did not feel that I had received enough support. Today*,* I see it more as having to take responsibility for my own life and not be upset that others eventually returned to their own lives*.” (Partner to the deceased, 55–64 years).

### Bereaved individuals’ experiences of support received or offered

The analysis resulted in five categories describing bereaved individuals’ experiences of support received or offered: managing grief together with close family and friends; supportive societal functions for the individual and the family; active personal efforts in grief management; professional support and treatment within healthcare; and no support received.

#### Managing grief together with close family and friends

A large number of participants reported receiving support from their family and friends, such as conversations, physical closeness, and practical assistance from one or several individuals. The results showed that support may come from many different sources within the social sphere, including from family members, colleagues, neighbours, and acquaintances of the bereaved person, and that the received support from social networks vary considerably between individuals. Many participants emphasised the importance of their family and friends. One participant wrote: “*I probably cannot put into words the responsibility and support I have received from my mother based on what happened.”* (Child to the deceased, 35–44 years). Some participants indicated that the support they received had been insufficient, such as: “*A little from family and friends in the beginning. But then it feels like people around me think you should get over the grief*.” (Partner to the deceased, 35–44 years), or “*One occasional friend who called and checked how I was doing*,* for example on difficult holidays/days. But otherwise not very much*.” (Partner to the deceased, 35–44 years).

#### Supportive societal functions for the individual and the family

Support from society was predominantly described as coming from the church, various types of peer support groups, and general counselling related to grief and coping with the loss. Supportive efforts from the church were reported to range from contact with specific professionals (e.g., priests, deacons), to conversations with someone within the church, or participation in church-based support groups, for example: “*Personal meetings/video calls with a deacon. Conversations with people in a church*.” (Child to the deceased, 55–64 years). It was also evident that the church is present at different locations in society. One participant wrote: *“I have attended regular counselling sessions at the Hospital Church and participated in a bereavement group through the Church of Sweden.*” (Partner to the deceased, 25–34 years).

Many respondents described participation in grief support groups and several different types of meetings. Both in-person meetings and digital groups were frequently mentioned, with other individuals who had undergone similar experiences, allowing the bereaved to feel understood in their emotions. One participant described it as “*The only context where you can fully be yourself*,* and everyone understands*.” (Parent to the deceased, 55–64 years).

Counselling as a form of support emerged in many different formats, such as group counselling, individual counselling, follow-up conversations, and calls to support-helplines. Access to crisis support was mentioned by several participants, including through insurance companies and the municipality. In addition to these sources, some participants also described support from existing institutions in their lives (e.g., workplace or educational system). The workplace was highlighted by several participants, for example: “*Understanding manager and employer*.” (Partner to the deceased, 45–54 years). One parent described the support their child received from preschool in the form of “*Extended hours at preschool for my older son*.” (Partner to the deceased, 25–34 years), whereas another participant, who was a student, instead described insufficient support from school: “*On the other hand*,* I did not really receive any support from the school. I was allowed to leave classes if I wanted to*,* but the problem then was that I fell behind*.” (Sibling to the deceased, 18–24 years).

#### Active personal efforts in grief management

The results indicated that bereaved individuals took active steps to improve their ability to cope with grief, and these efforts varied widely. Examples included participation in grief courses, reading books and texts about grief, or training to become a therapist, as well as involvement in various support groups and organisations. One participant described the impact of grief courses on their bereavement: “*I actively sought a lot of [support] myself; the grief course gave me a great deal*.” (Partner to the deceased, 35–44 years). One participant described their involvement in non-profit support organisations: “*I became involved in the association during the first years. I was responsible for the in-person sibling group and was part of the board*.” (Sibling to the deceased, 55–64 years). Several participants also described support in the form of séances and spiritual counselling; one individual wrote: “*My strong belief that [my child] exists in another dimension. I can communicate with him*.” (Parent to the deceased, 65–74 years). Self-medication through alcohol and food was also mentioned by a few. One participant wrote: *“I comforted myself with alcohol*,* and because of that I was not allowed to receive psychological treatment.”* (Parent to the deceased, 55–64 years).

#### Professional support and treatment within healthcare

Many participants mentioned contact and conversations with a psychologist, therapist, or counsellor, and many expressed that these contacts had been important to them. One participant wrote: “*When my brother died a few years later*,* I initially thought I would manage on my own*,* but after a month I chose to contact the primary care centre and thereby obtained contact with a psychologist*,* which was invaluable in my grieving process*.” (Sibling to the deceased, 35–44 years).

Contact with physicians involving medication prescriptions for physical symptoms and sick leave was mentioned by several participants. Sick leave was described in several cases as complicated to obtain in a satisfactory manner. One participant wrote: *“My general practitioner saw the need for a rehabilitation coordinator since my manager wanted me to perform work tasks that my working hours did not allow for during sick leave.”* (Partner to the deceased, 55–64 years).

Support from the healthcare unit that had cared for the deceased, both in connection with the death and afterwards, was described by several participants. One parent wrote: *“We have received fantastic support. The healthcare services that were there for our daughter while she was alive have rallied around us and been there for us.”* (Parent to the deceased, 45–54 years).

#### No support received

The results also showed that it was relatively common for participants to report having received no support at all. One participant wrote: “*Not much support at all*,* neither from friends nor from healthcare.”* (Sibling to the deceased, 25–34 years). The majority of participants who mentioned a lack of support did not elaborate on why they had not received support, while some described that they did not need support, that they had not sought support, that they had been denied the support they sought, or that they had not been receptive to the support offered to them. One participant wrote: *“I did not feel that I received much support*,* at least not in the beginning. The primary care centre did not even want to offer me a doctor’s appointment when I made initial contact.”* (Partner to the deceased, 25–34 years).

### Support that the bereaved wished they had received

The analysis resulted in six categories describing support that the bereaved wished they had received: information for the bereaved; expanded bereavement support through human interaction; organisation of support with consideration of temporal aspects; access to treatments for grief and grief-related disorders; practical support in everyday life; and increased knowledge about grief in society.

#### Information for the bereaved

Many participants wished for improved information about available bereavement support within healthcare, municipalities, workplaces, and other institutions. Participants reported a lack of information about grief from healthcare services, as well as information regarding the death from the treating healthcare unit. For example, one participant expressed a need to “*Be informed that the doctor made the right decision to discontinue the ventilator*.” (Partner to the deceased, 45–54 years). One participant mentioned a need for written information at the time of the death, as grief and shock made it difficult to remember verbal information.

Several participants raised the need for information and guidance regarding the management of the estate of the deceased, with one participant writing: “*A lot of the information was unclear or contradictory to other information. It was not pleasant to encounter unclear answers during a difficult and intense period when*,* as a next of kin*,* I was given full responsibility for the estate*.” (Child to the deceased, 25–34 years).

#### Expanded bereavement support through human interaction

It was common for participants to express an increased need for conversations about the deceased and the death, as well as about their own grief experiences, emotions, and well-being. Participants also reported a need for support in managing their grief. Contact with individuals who are knowledgeable and experienced in grief was mentioned by many participants, including various healthcare professionals (e.g., psychologists, counsellors, physicians), but also individuals outside the healthcare system (e.g., representatives of the church, the municipality, family members, friends).

There was a clear desire for support specifically tailored to different groups of bereaved individuals, partly depending on how the loved one had died (e.g., suicide, substance use), and partly depending on the relationship to the deceased (e.g., child, parent, sibling). One participant wrote: “*We would have wished for family support for our children; they were left completely without any opportunity to talk about the grief*.” (Parent to the deceased, 55–64 years). The need for specialized support for different types of individuals was also addressed; for example, one participant highlighted the lack of support for people with neurodevelopmental disorders: *“Help with grief processing for autistic people. As an autistic person*,* processing takes much longer than ‘normal*,*’ but healthcare does not take this into account at all.”* (Sibling to the deceased, 35–44 years). Some participants also raised the lack of support for other relatives of the bereaved than the immediate family, with one participant writing: *“I perhaps wish that healthcare could offer a psychologist or therapy. Maybe also for my partner*,* who is greatly affected by my grief and would need help.”* (Sibling to the deceased, 25–34 years).

#### Organisation of support with consideration of temporal aspects

Several participants expressed a need for a greater amount of support or support provided over a longer period of time. Continuity, organisation, and regularity of support were mentioned repeatedly. One participant stated *“That there was someone who maintained continuous contact with me. Many people talk during the first year*,* but my worst downturns have come later*,* and now it is five years since [the death]. One needs support for many years to come.”* (Parent to the deceased, 45–54 years). The lack of centralised expertise was also highlighted: “*That there was centralised expertise*,* so you did not have to search for help yourself*,* for the children and me.”* (Partner to the deceased, 45–54 years).

Temporal aspects that were mentioned included a desire for follow-up from healthcare services as well as more immediate support in connection with the death. Some participants raised shortcomings in referrals to adult psychiatry or primary care after the termination of contact with child and adolescent services. One participant wrote: *“In my contact with child health services*,* I see that they have forms to identify various risks (e.g.*,* intimate partner violence*,* depression*,* stress). For example*,* I have expressed stress and low mood there*,* but my feeling is that they did not have the right context to assess my grief.”* (Partner to the deceased, 35–44 years).

A desire for outreach-based support, so that bereaved individuals do not have to actively seek support themselves, was expressed by several participants, partly because they do not have the energy or the knowledge of where to find support. One participant described it: *“When you are in crisis*,* it is difficult to have the energy to seek help on your own. It should be easier to be offered support without having to ask for it yourself*,* for example*,* during medical consultations about sick leave*.” (Sibling to the deceased, 25–34 years).

#### Access to treatments for grief and grief-related disorders

The results showed that participants wished for various forms of therapy such as “*psychotherapy*”, “*behavioural activation*”, “*PTSD (posttraumatic stress disorder) treatment*” and “*trauma therapy*”. In addition to therapy, there was a need for pharmacological treatment for sleep problems, pain, and anxiety. One participant wrote, *“Help (Propavan) to get my sleep in order. It took 10 years before a doctor helped me to sleep. That I have now taken Propavan for 1.5 years and been able to sleep has contributed to less anxiety. However*,* I would gladly receive help for the PTSD and the panic feelings that I sometimes experience*.” (Partner to the deceased, 45–54 years). An unspecific need for other forms of healthcare support beyond medication also emerged, with one participant writing *“When you seek healthcare after a loss*,* there should be more help available than medication; my son died of an overdose*,* and they want to give me pills*,* it’s absurd*.” (Parent to the deceased, 55–64 years).

#### Practical support in everyday life

The results show that several participants had a need for practical support in everyday life, such as childcare support from family and friends or extended approved leave or sick leave from work. Supportive measures from schools were also mentioned: *“I did not want to fall behind in school*,* so it was a matter of just pushing the grief aside and carrying on. (Something I perhaps regret a bit today.) I wish there had been some other way the school could have helped.”* (Sibling to the deceased, 18–24 years).

Practical and financial support in managing the estate of the deceased and funeral arrangements were expressed by several participants: *“Around all the practical matters that have to be arranged. A lot of legal issues you do not understand that are simultaneously tied to the emotional aspects. Choosing a burial plot*,* a gravestone (indirectly my own) was tough.”* (Partner to the deceased, 45–54 years). Financial support also included the right to a greater number of subsidised or free healthcare visits (e.g., psychologist visits, therapy). One participant wrote: *“It is costly to pay for therapy myself*,* and I had to stop because of that.”* (Partner to the deceased, 45–54 years).

#### Increased knowledge about grief in society

Several participants expressed a desire for better and more extensive knowledge about grief within healthcare and across various societal institutions. For example, one participant noted that they *“also wished that the physician I met had shown more compassion or had more knowledge specifically about grief.”* (Partner to the deceased, 35–44 years), while another participant wished that *“more people knew how to talk to children who are experiencing grief.”* (Grandchild to the deceased, 25–34 years). One participant suggested the introduction of education on death and grief within compulsory school as a way to prevent destructive thoughts during grief.

In addition to increased knowledge, participants also called for greater understanding of the grief process from family and friends, workplaces, the educational system, and healthcare services. One participant described a need for *“greater awareness that the world continues to stand still for the affected person long after the funeral has taken place*,* that people know this and can be helped to understand that support is needed for more than two months after the death.”* (Partner, 45–54 years), and another wished for *“a more open attitude from society*,* allowing people to grieve for as long as it takes.”* (Sibling to the deceased, 35–44 years). Validation of one’s grief also emerged in the results, with one participant writing: *“Acknowledge my grief; do not be surprised that I am still unwell. The acute help was good*,* but then people grow tired.”* (Partner to the deceased, 35–44 years).

## Discussion

This study investigated perceived and desired support in bereaved individuals following the death of a loved one from a broad perspective, without considering cause of death, time since the death, or severity of grief. Support was found to originate from many different sectors of society, ranging from family, friends, the church, and non-profit support groups to primary care and specialist healthcare. The support received included, among other things, conversational support, practical assistance in everyday life, and treatment of grief-related complications. Active personal grief processing was also reported as components of bereavement support, for example, reading about grief and participating in grief courses. However, a lack of support was also identified, and nearly 60% of participants perceived the support they had received as insufficient. Desired support included information, understanding from the surrounding environment, increased knowledge about grief in society, as well as financial and practical assistance in everyday life and in managing the estate of the deceased. Wishes were also expressed for changes in the design of bereavement support regarding who receives support, how support is structured, and how much support a bereaved person is entitled to, as well as for specialised support from healthcare through counselling, therapy, and pharmacological treatment.

The perceived bereavement support in this study corresponds well with literature from other parts of the world, such as a recent international systematic review on how bereavement support is structured within healthcare [9], and with the bereavement support pyramid developed by the Irish Hospice Foundation [19]. It also aligns with recent Swedish research in a palliative care context, which similarly suggests that bereavement support extends beyond professional services and may include informal, community-based, and practical forms of support [26]. In line with these models, participants in the present study described support at several levels, including informal support from family, friends, colleagues, and other social networks; organised support from civil society, the church, and peer-support groups; and more specialised support from healthcare, such as counselling, psychological treatment, medication, and sick leave. The findings also correspond with previous literature in showing that bereavement support is not provided by one single actor, but is distributed across informal, community-based, and professional systems. At the same time, the present study adds to this literature by showing that bereaved individuals in Sweden experience gaps in the visibility, continuity, and organisation of such support.

The findings have implications for how bereaved individuals with more complex grief-related needs may be identified and supported. Several participants described a need for specialised healthcare support, including psychological treatment, medication for sleep problems and anxiety, and long-term follow-up. These findings suggest that some bereaved individuals may require more targeted professional support, even though most bereaved people do not need specialised treatment [19, 20]. With the introduction of ICD-11 in Sweden, PGD will be more clearly defined through explicit diagnostic criteria, which may facilitate the identification of bereaved individuals in need of specialised bereavement support and potentially increase their access to appropriate care [6, 22]. However, determining what support should be offered to bereaved individuals may remain challenging, as bereaved individuals constitute a highly heterogeneous group with a wide range of support needs, as shown in the results of this study.

The Swedish healthcare system is responsible for providing initial bereavement support for most bereaved individuals following a death, including enabling relatives to say farewell to the deceased and offering an explanation of the death [10]. Previous Swedish research in end-of-life care has also shown that follow-up conversations after the death may be valued by bereaved family members, particularly when they provide an opportunity to ask questions and receive explanations about the illness and death. However, not all bereaved family members receive such follow-up, even when they would have wanted it [27]. After the initial phase, there are no clear regulations governing ongoing bereavement support within healthcare or across other institutions in Sweden.

There are differing opinions regarding when healthcare should intervene in the grieving process. Although support may be offered through counselling by therapists, counsellors, or psychologists, such services are not always clearly delineated from specialised bereavement care, thereby complicating when or whether healthcare should intervene. Concerns have also been raised that increased healthcare involvement may contribute to over-pathologisation of grief and contribute to a general perception that grief does not belong to everyday life but is something that often requires treatment [28, 29]. In addition to the risk of pathologising grief, healthcare also faces challenges in identifying bereaved individuals, as there is no consistent definition of which relatives are the closest bereaved, who will require additional support, or who will manage without healthcare involvement. Without the ability to identify who is entitled to support, it is difficult to establish standardised guidelines for bereavement support.

An important factor is also whether bereaved individuals perceive the support as helpful. A study conducted in Australia reported that support from healthcare was more often perceived as unhelpful. In contrast, informal support, such as from family or funeral directors, more often facilitated the grieving process [20]. Bereavement support as primary prevention, has been shown to be more effective when the bereaved individual seeks support themselves, both in terms of personal suffering and economic cost, compared with healthcare offering support to all bereaved individuals [2]. This aligns with the results of the present study, as nearly half of the participants did not perceive a need for additional support, and some felt they did not need any support at all. Previous research has examined the effects of bereavement support in the form of brief follow-up interventions from healthcare, such as informational leaflets or a telephone call. It concluded that such interventions help bereaved individuals feel acknowledged and gain access to information, but showed no effect on their general well-being [30].

Regardless of the needs for bereavement support identified by bereaved individuals, those who provide support must possess appropriate knowledge as well as the opportunity and resources to deliver support effectively. While the personal social network is a significant source of support for the bereaved, the public sector (e.g., healthcare services, public health authorities, social services) has a responsibility to ensure that evidence-based and accessible information about bereavement is available. This includes information that acknowledges individual variation in grief, while also clarifying common reactions, signs of elevated risk (e.g., PGD), when and where to seek additional support, as well as practical guidance for family and friends on supportive responses, and on how to facilitate access to services when needed. The role of civil society is difficult to regulate. Still, structured communication and collaboration between civil society organisations and the public sector could enable more bereaved individuals in need of professional support to actually receive it, for example, through shared referral pathways, clear signposting, and consistent information.

Currently, healthcare institutions lack sufficient knowledge regarding how grief should be evaluated and subsequently supported [18], which makes it difficult to establish patient-centred and evidence-based national guidelines for the management of bereaved individuals. Nonetheless, the recognition of PGD as a formal diagnosis and the subsequent increase in research are promising for the development of clinical guidelines, although gaps remain.

### Limitations and strengths of the study

This study had some limitations. Questions were administered via a web-based survey, which made it impossible to clarify probable misunderstandings or ask follow-up questions to participants who may have misunderstood a question or responded to something other than intended. The level of detail in the responses varied, with some brief or less specific responses. This made some parts of the analysis more interpretive, particularly when coding short responses with limited contextual information. To address this, the analysis was conducted as close to the participants’ own wording as possible, and codes and categories were discussed between authors to strengthen credibility. Perceptions of received and desired support may change over time following a death, as demonstrated in this study, which complicates the interpretation of the results. Time since loss varied substantially among participants, as there was no time restriction regarding when the death occurred for inclusion in the study. Research on grief and the development of bereavement support has evolved considerably in recent years, and this study did not account for when the loved one died and thus when participants accessed bereavement support. This represents a limitation of the analysis, as it introduces a risk that some findings reflect outdated information that may no longer be relevant, for example, regarding wishes for digitally available support, which has developed substantially in recent years. The study population showed good variation in age and educational level, but women were overrepresented. This creates uncertainty regarding whether the results are applicable to men, particularly given evidence suggesting that men in Sweden have different bereavement support needs than women [31]. The sample showed good variation in relationship to the deceased; however, relatives of individuals who died by suicide may be considered overrepresented. Slightly more than 1% of deaths in Sweden in 2024 were due to suicide [32], whereas about 26% of participants had lost a loved one to suicide. Grief following suicide may be considered more traumatic and stigmatised than grief following other causes of death and may therefore have influenced the results toward greater use of and desire for specialised healthcare support.

The study also has important strengths. The relatively large sample of bereaved participants provided a broad empirical basis for the analysis and made it possible to capture a wide range of experiences of bereavement support in Sweden. The variation in participant characteristics and loss-related circumstances strengthened the breadth of the material and allowed the study to reflect support experiences across different bereavement contexts. Another strength is that the study included questions about both received and desired support, which enabled identification of not only existing support sources but also perceived gaps and unmet needs. The use of open-ended questions made it possible for participants to describe support in their own words, which is valuable in a field where knowledge about how support should be organised remains limited. Finally, the inductive qualitative content analysis, the discussions between authors throughout the analytic process, and the inclusion of representative quotations strengthened the credibility and transparency of the findings.

## Conclusion

The results demonstrate that different types of bereavement support in Sweden are provided by a wide range of societal actors, from close family and friends to civil society and the public sector. However, the findings also suggest that there is a need for improved structure, continuity, and visibility of available support, as well as recognition that bereaved individuals may need different types of support. Currently, there is limited evidence on which forms of bereavement support are effective; however, this study contributes to highlighting important gaps in the bereavement support currently offered in Sweden. Further research is needed to determine which bereaved individuals require specific types of support, and at what point in time, in order to target interventions appropriately while also ensuring that bereavement support is both effective and socioeconomically sustainable.

## Data Availability

Data are available from the authors upon reasonable request.

## Abbreviations

DSM-5-TR: Diagnostic and Statistical Manual of Mental Disorders, Fifth Edition, Text Revision
ICD-11: International Classification of Diseases 11th Revision
PGD: Prolonged grief disorder
PTSD: Posttraumatic stress disorder
